# Validation and application of the Dermatology Life Quality Index score, a modification of the DLQI score, in psoriasis patients

**DOI:** 10.1186/s41043-024-00587-3

**Published:** 2024-06-22

**Authors:** Qin Zou, Yibo Luo, Dan Hao, Mengmeng Li, Chen Jihui

**Affiliations:** 1 Department of Dermatology, West China Hospital of Sichuan University, No.37 Guoxue Alley, Wuhou District, Chengdu City, Sichuan Province 610041 PR China; 2 West China School of Nursing, Sichuan University, No.37 Guoxue Alley, Wuhou District, Chengdu City, Sichuan Province 610041 PR China

**Keywords:** Dermatology Life Quality Index, Psoriasis, Questionnaire modifications, Validation and application

## Abstract

**Background:**

All the scoring methods for the DLQI miss the moderate impact of the disease on patients, which may underestimate the impact of psoriasis on patients’ quality of life. To improve the accuracy of the assessment of the Dermatology Life Quality Index score (DLQI) for patients with psoriasis, this study proposed and validated a new scoring method, the DLQI-NS, which includes the moderate impact option in the self-assessment of each item in psoriasis patients.

**Methods:**

A cross-sectional study was conducted in which patients with psoriasis were enrolled. A total of 425 participants completed the DLQI, DLQI-NS and Skindex-16 questionnaires. Reliability, validity, ceiling and floor effects were evaluated of both DLQI and DLQI-NS questionnaires.

**Results:**

About 14.4-32.5% of the patients reported a moderate impact on quality of life. The DLQI-NS allowed 17 more patients (4.0%) to achieve severe disease. The Cronbach’s alpha coefficient of the DLQI-NS was 0.90, and that of the DLQI was 0.89. The KMO test results for the DLQI-NS and DLQI were 0.927 and 0.916, respectively. One factor was identified for each questionnaire. The items of the DLQI-NS showed an item-total correlation from 0.52 to 0.82, and the DLQI questionnaire’s item-total correlation ranged from 0.47 to 0.83. The DLQI-NS, DLQI total score and Skindex-16 had Spearman’s rank correlation coefficients of 0.89 and 0.84, respectively. Both the DLQI-NS and DLQI showed significant moderate correlations with the BSA (0.51 vs. 0.50) and PASI (0.47 vs. 0.46). No ceiling effects were observed for any of the items of both questionnaires.

**Conclusion:**

The validity and reliability of the DLQI-NS and DLQI were good, but the DLQI-NS was superior to the DLQI. The DLQI-NS is an effective self-assessment tool for assessing quality of life in psoriasis patients.

## Introduction

Psoriasis is rarely life-threatening but multifaceted skin disorder that can seriously affect the quality of life (QOL) of patients, including their social relationships, daily activities and other aspects of life [1, 2]. It is estimated to affect more than 60 million people worldwide [3]. To date, there is no definitive cure for the disease, and, therefore, patients usually need long-term treatment to control symptoms and improve quality of life. Several guidelines recommend the assessment of health-related quality of life (HRQoL) as an outcome measure for the effectiveness of psoriasis treatment [4–8]. There are several types of HRQoL measurement scales for psoriasis patients, involving disease-specific measures and generic tools [6]. The generic scales used include the Short-Form Health Survey Questionnaire (SF-36), the World Health Organization Quality of Life Scale (WHOQOL), the EuroQol Five Dimensions Questionnaire (EQ-5D), and the Nottingham Health Profile (NHP) [2, 9–11]. Skin disease-specific instruments, such as the Skindex-16, Dermatology Life Quality Index (DLQI), and Dermatology Life Quality Scale (DQOLS), are considered more relevant and therefore preferred by patients [12, 13].

The DLQI is the most frequently applied questionnaire for assessing HRQoL in dermatology [12], which has been used in more than 40 skin diseases and has been translated into more than 100 languages [14]. Given its simplicity of use and ability to detect even small differences in a patient’s health status, DLQI is well integrated into the clinical practice in dermatology and recognized by dermatological professional associations. Many clinical guidelines for the management of psoriasis involve DLQI (score range 0–3), such as clinical decisions, admissions and discharge [6, 15, 16]. Along with the Psoriasis Area and Severity Index (PASI), it was identified as the recommended endpoint and core outcomes for therapeutic efficacy in clinical trials worldwide [17–19].

According to the original scoring system, four distinct response options (‘not at all’, ‘a little’, ‘a lot’, and ‘very much’) are attached to ten items of the questionnaire to determine the extent to which their skin disease affects each of their lives [12]. To achieve more appropriate, valid, sensitive, and reliable assessment, there have been continuous discussions concerning DLQI modifications in psoriasis, including scoring, item selection, recall period, disease, symptom, body part, and illustration changes [14]. (1)Item modifications. First, to achieve a more accurate assessment of quality of life, new items are added or certain items are removed from the original DLQI questionnaire. Second, modifications were made to the existing items. (2)Revision of recall period. Modifications that lengthen or shorten the DLQI recall period. (3)The target population was Changed. The DLQI questionnaire was applied to other diseases or age groups. (4) Modifications of response or scoring methods. Rencz F et al. proposed a new scoring formula to adjust the total DLQI score of patients for the number of ‘not relevant’ responses (NRRs), which showed good validity and can improve patients’ access to biologics [20, 21].

However, all the scoring methods of the DLQI ignore the moderate impact of the disease on patients, especially in the aspects of symptoms and feeling, work and school, personal relationships and treatment [14]. Therefore, valuable patients’ information may be discarded. This may underestimate the impact of psoriasis on patients’ quality of life, which in turn affects some clinical decisions in the management of psoriasis. To improve the accuracy of patients’ self-evaluation scores, this study proposed a new scoring method, the DLQI-NS, which includes the moderate impact option in the self-assessment of each item in psoriasis patients. Aimed to evaluate the informativity and sensitivity of DLQI-NS, a cross-sectional study was conducted in psoriasis patients subjected to DLQI, DLQI-NS and Skindex-16 questionnaires.

## Methods

### Study design and patient population

Between May 2021 and December 2022, a cross-sectional questionnaire survey was administered at the Department of Dermatovenereology, West China Hospital of Sichuan University. A total of 430 patients were recruited.

### Inclusion and exclusion criteria

Patients who met the following criteria were included: (1) were aged more than 18 years with no sex restriction, (2) were diagnosed with psoriasis by dermatologists according to the psoriasis area and severity index (PASI) score and body surface area (BSA) score, and (3) could read or write Chinese independently. Participants with the following conditions were excluded: (1) had a malignant tumor; (2) had serious dysfunction of the liver, kidney or other organs; or (3) were pregnant or lactating.

### Data collection

Eligible patients were invited to complete the questionnaires on the first day of admission. Quality of life was assessed by the DLQI, DLQI-NS and Skindex-16 questionnaires, and the clinical severity of psoriasis was assessed by using PASI score and BSA score. It took the participants 5–10 min to complete the questionnaires. A total of 425 patients completed all the qualifying questionnaires and thirty-six patients completed in the DLQI-NS and DLQI questionnaires again two weeks later.

### Outcome measures

#### DLQI and DLQI-NS

The DLQI is designed to assess HRQoL in skin disease patients [14]. The ten-item questionnaire scores quality of life impairment due to the dermatologic condition, including aspects such as symptoms, side effects of treatment, daily activities, work or school, personal relationships, leisure activities, and feelings of embarrassment. Four distinct scores may be assigned to all items of the questionnaire regardless of the number of response options for that item: ‘not at all’ or ‘not relevant’, 0; ‘a little’, 1; ‘a lot’, 2; and ‘very much’, 3. The DLQI total score is calculated by summing the scores of the 10 items ranging from ‘0’ to ‘30’, where higher scores indicate greater disability experienced by patients. The overall impact of skin disease on a patient’s HRQoL according to the DLQI scale is described as follows: a total score greater than 21 indicates an extremely large effect, 11–20 = very large effect, 6–10 = moderate effect, 2–5 = small effect, and 0–1 = no effect [5].

The DLQI new scoring method (DLQI-NS) adds a moderate effect option to all items based on the original scale. The 10 items of the scale are rated on a 5-point scale (“not at all” or “not relevant” =0, “a little” =1, “moderate” =2, “a lot” = 3 and “very much” = 4), yielding a total score from 0 to 40. Higher total scores represent greater impairment of one’s quality of life.

#### Skindex-16

The Skindex-16 covers 3 dimensions: symptoms, emotions and functions. It contains 16 items, each rated on a 7-point Likert scale ranging from “never bothered” to “always bothered”. All item scores are transformed to a linear scale from 0 to 100, and the total score is taken as the average of patient responses to each dimension scale [12, 22].

#### PASI and BSA

Dermatologists examined all patients and assessed the severity of psoriasis using PASI and BSA scores [23]. The PASI assesses the degree of desquamation, infiltration and erythema of the patient’s lesion. These features were assessed using a four-point scale: very marked symptoms (4), marked symptoms (3), moderate symptoms (2), slight symptoms (1), and no symptoms (0). The PASI takes both severity and coverage into consideration. The BSA is the arithmetic average of the affected skin surface; the total body surface area is divided into 10% for the head, 20% for the upper extremities, 30% for the trunk, and 40% for the lower extremities. The surface area of a patient’s palm is 1%.

#### NRS

The NRS is a simple tool that has been validated for measuring pruritus and pain [24]. It is used to assess pruritus in patients with psoriasis in this study. It is a graphic tool with a number ranging from 0 to 10; patients mark 0 to represent no symptoms, and 10 to represent the worst imaginable symptoms [24].

### Ethical consideration

This study was approved by the West China Hospital of Sichuan University Biomedical Research Ethics Committee (No. 2021.545). Informed consent was obtained from all patients before enrollment in the study.

### Statistical analysis

The statistical analysis was performed with SPSS 26.0 (Chicago, IL, USA). Continuous variables are expressed as the mean ± standard deviation (SD), while categorical variables are expressed as proportions (%). The reliability, validity, ceiling and floor effects between the DLQI-NS and the DLQI questionnaire were compared. All the applied statistics were two-sided with a significance level of *p*<0.05.

The reliability of both questionnaires was evaluated by internal consistency, split-half reliability and test–retest reliability. Cronbach’s alpha was used to assess internal consistency. Test–retest reliability was evaluated by the Spearman rank correlation coefficient for scores obtained at baseline and two weeks later. Split-half reliability was assessed using Guttman’s half coefficient and the Spearman-Brown coefficient.

Validity was tested as construct validity, criterion validity and convergent validity. Construct validity was assessed by factor analysis. The Kaiser‒Meyer‒Olkin (KMO) and Bartlett tests were used to assess the feasibility of performing the factor analysis. Construct validity was also measured by Spearman’s rank correlation analysis between DLQI-NS item scores and total scores. To assess criteria validity, the correlation between the DLQI-NS and Skindex-16 questionnaire scores was analyzed with Spearman’s rank correlation analysis. Convergent validity was tested by Spearman’s rank correlation analysis between the DLQI-NS score and the PASI and BSA scores.

The ceiling and floor effects of the questionnaire total or item scores were assessed by measuring the proportion of the highest and lowest scores of the DLQI and DLQI-NS, respectively. They were considered present if > 15% of patients had the highest or lowest possible score.

## Results

### Patient characteristics

Overall, 430 adult psoriasis patients were invited to participate in this study. Finally, 425 participants (98.84%) completed the questionnaire. Patient characteristics are described in Table 1. The mean age was 41.86 ± 14.78 years (minimum 18 years, maximum 84 years). More than half of the participants were male (*n* = 278, 65.4%). The duration of disease was 9.62 ± 8.41 years (minimum, one month; maximum, 50 years). The mean BSA and PSAI scores were 21.56 ± 13.37 and 15.96 ± 7.73, respectively. The average scores of quality of life tools were 13.19 ± 8.02 for DLQI-NS, 11.24 ± 6.06 for DLQI and 26.46 ± 14.47for Skindex-16, respectively.

**Table 1:** Characteristics of patients with psoriasis (*N* = 425)

Items	*n*, %, Mean ± SD, Range
Sex	
Male	278(65.4)
Female	147 (34.6)
Marriage	
Unmarried	98 (23.1)
Married	306 (72.0)
Divorced	19 (4.5)
Widowed	2 (0.5)
Education background	
Master or above	17(4.0)
Bachelor degree	91(21.4)
College degree	84 (19.8)
Secondary education	185 (43.5)
Primary school	48(11.3)
Age (year)	41.86 ± 14.78 (18–84)
Duration of disease (year)	9.62 ± 8.41(0–50)
BSA (%)	21.56 ± 13.37(1–78)
PASI	15.96 ± 7.73(3.5–48)
NRS	1.31 ± 1.94(0–8)
DLQI-NS	13.19 ± 8.02(0–40)
DLQI	11.24 ± 6.06(0–30)
Skindex-16	26.46 ± 14.47(0.79–74.41)

### The results of the DLQI-NS and DLQI scores

**Fig. 1:**
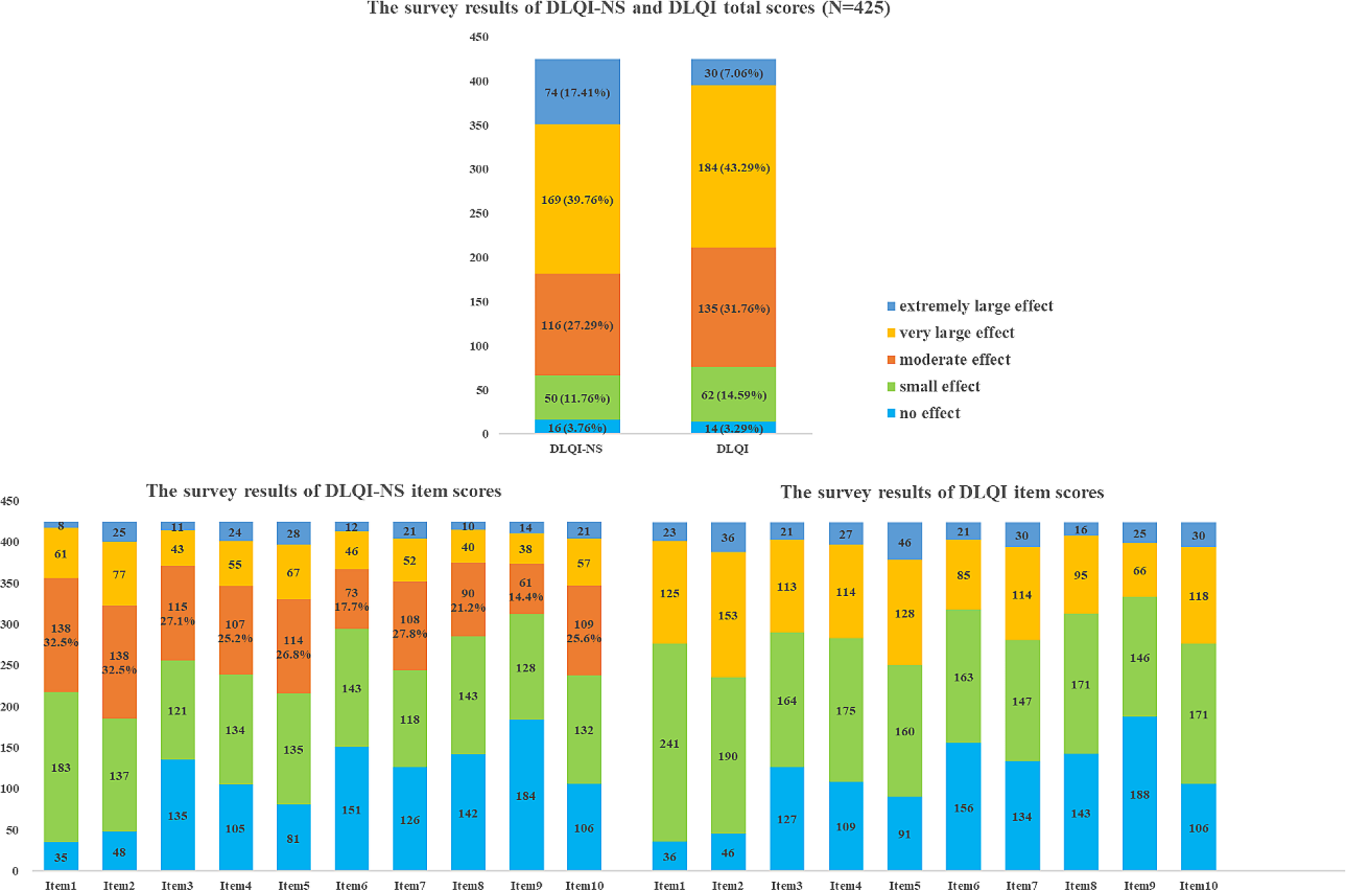
The results of DLQI-NS and DLQI scores

In the total samples, mean DLQI-NS and DLQI scores were 13.19 ± 8.02 and 11.24 ± 6.06, respectively, which means that DLQI-NS has slightly raised the the score compared to the original DLQI. The proportions of DLQI and DLQI-NS scores according to Hongbo’s bands are presented in Fig. 1. According to the criteria for clinical classification of psoriasis severity [8], the DLQI-NS allowed 17 more patients (4.0%) to achieve severe disease (PASI > 10 or BSA > 10% and DLQI > 10).

### Reliability

The Cronbach’s alpha coefficient of the DLQI-NS was 0.90, and that of the DLQI was 0.89. Test–retest reliability was assessed by asking participants (*n* = 36) to complete the 2 questionnaires twice, 2 weeks apart. The intraclass correlation coefficients of DLQI-NS and DLQI were 0.72–0.95 and 0.78–0.98, respectively. The Spearman-Brown coefficient and Guttman’s half coefficient of the DLQI-NS and DLQI were 0.88 and 0.86, respectively (Table 2).

**Table 2:** The reliability of DLQI-NS and DLQI

	DLQI-NS(*N* = 425)					DLQI(*N* = 425)			
	Cronbach’s a	Spearman rank correlation coefficient(*n* = 36)	Spearman-Brown coefficients	Guttman’s half coefficients		Cronbach’s a	Spearman rank correlation coefficient(*n* = 36)	Spearman-Brown coefficients	Guttman’s half coefficients
Item1	—	0.72^**^	—	—		—	0.78^**^	—	—
Item2	—	0.87^**^	—	—		—	0.78^**^	—	—
Item3	—	0.86^**^	—	—		—	0.85^**^	—	—
Item4	—	0.83^**^	—	—		—	0.87^**^	—	—
Item5	—	0.83^**^	—	—		—	0.89^**^	—	—
Item6	—	0.81^**^	—	—		—	0.83^**^	—	—
Item7	—	0.91^**^	—	—		—	0.81^**^	—	—
Item8	—	0.94^**^	—	—		—	0.82^**^	—	—
Item9	—	0.90^**^	—	—		—	0.95^**^	—	—
Item10	—	0.82^**^	—	—		—	0.87^**^	—	—
Total score	0.90	0.96^**^	0.88	0.86		0.89	0.98^**^	0.88	0.86

**correlation is significant at the 0.01 level (2-tailed)

### Validity

Construct validity was assessed by factor analysis and Spearman’s rank correlation analysis between items and total scores on the DLQI-NS and DLQI. The KMO test results for the DLQI-NS and DLQI were 0.927 and 0.916, respectively. Bartlett’s sphericity test revealed chi-square values of 1995.288 and 1758.842 (*p* < 0.001) for the questionnaires. The results showed that factor analysis was suitable for both questionnaires. One factor was identified for each questionnaire, accounting for 53.36% of the variance in the DLQI-NS and 49.85% of that in the DLQI questionnaire. The results showed that the construction scores on both scales were good, but the DLQI-NS was superior to the DLQI. All the items of the DLQI-NS and DLQI had good item-total correlations. The DLQI-NS showed an item-total correlation from 0.52 to 0.82, and the DLQI questionnaire’s item-total correlation was 0.47–0.83 (Table 3).

**Table 3:** The item-total correlation of DLQI-NS and DLQI (*N* = 425)

DLQI-NS total score			DLQI total score	
	Spearman rank correlation coefficient			Spearman rank correlation coefficient
Item1	0.52^**^		Item1	0.47^**^
Item2	0.71^**^		Item2	0.69^**^
Item3	0.67^**^		Item3	0.78^**^
Item4	0.69^**^		Item4	0.70^**^
Item5	0.82^**^		Item5	0.83^**^
Item6	0.66^**^		Item6	0.71^**^
Item7	0.73^**^		Item7	0.76^**^
Item8	0.75^**^		Item8	0.73^**^
Item9	0.68^**^		Item9	0.69^**^
Item10	0.71^**^		Item10	0.66^**^

Note. **=correlation is significant at the 0.01 level (2-tailed)

Regarding criterion validity, the DLQI-NS, DLQI total score and Skindex-16 were strongly correlated. Spearman’s rank correlation coefficients were 0.89 and 0.84, which were statistically significant (Table 4).

**Table 4:** Spearman’s rank correlation coefficients between Dermatology Life Quality Index questionnaires and Skindex-16

	Skindex-16			
	Emotions	symptoms	functions	Total score
**DLQI-NS**				
Symptoms and feelings	0.43^**^	0.70^**^	0.65^**^	0.69^**^
Daily activities	0.52^**^	0.60^**^	0.75^**^	0.71^**^
Leisure	0.57^**^	0.66^**^	0.81^**^	0.79^**^
Work and school	0.46^**^	0.58^**^	0.72^**^	0.68^**^
Personal relationship	0.46^**^	0.66^**^	0.73^**^	0.73^**^
Treatment	0.47^**^	0.51^**^	0.62^**^	0.62^**^
Total score	0.61^**^	0.76^**^	0.91^**^	**0.89** ^**^
**DLQI**				
Symptoms and feelings	0.38^**^	0.66^**^	0.57^**^	0.62^**^
Daily activities	0.48^**^	0.57^**^	0.70^**^	0.67^**^
Leisure	0.51^**^	0.63^**^	0.74^**^	0.72^**^
Work and school	0.40^**^	0.53^**^	0.63^**^	0.60^**^
Personal relationship	0.42^**^	0.61^**^	0.69^**^	0.68^**^
Treatment	0.43^**^	0.46^**^	0.55^**^	0.55^**^
Total score	0.55^**^	0.75^**^	0.88^**^	**0.84** ^**^

Note. **=correlation is significant at the 0.01 level (2-tailed)

The convergent validity of both scales was assessed by Spearman’s rank order correlations with the BSA, PASI and NRS scores. Both the DLQI-NS and DLQI showed significant moderate correlations with the BSA (0.51 vs. 0.50) and PASI (0.47 vs. 0.46). It is obvious that the DLQI-NS correlated slightly better with the BSA and PASI (0.51 vs. 0.47). Neither questionnaire was correlated with the NRS score (0.00 vs. 0.02).

### Floor and ceiling effects

The results indicated that the DLQI-NS total scores ranged from 0 to 40, and the DLQI total scores ranged from 0 to 30. The proportions of respondents with the lowest values for items 1 (8.2% or 8.5%) and 2 (11.3% or 10.8%) were less than 15%, indicating that there were no floor effects. No ceiling effects were found for any of the items of either the DLQI-NS or the DLQI. Interestingly, the floor effects were the same for both questionnaires (Table 5).

**Table 5:** Floor and ceiling effects of DLQI-NS and DLQI

	FE, *n* (%)			CE, *n* (%)	
	DLQI-NS	DLQI		DLQI-NS	DLQI
Item1	35(8.2)	36 (8.5)		8(1.9)	23(5.4)
Item2	48(11.3)	46(10.8)		25(5.9)	36(8.5)
Item3	135(31.8)	127(29.9)		11(2.6)	21(4.9)
Item4	105(24.7)	109(25.6)		24(5.6)	27(6.4)
Item5	81(19.1)	91(21.4)		28(6.6)	46(10.8)
Item6	151(35.5)	156(36.7)		12(2.8)	21(4.9)
Item7	126(29.6)	134(31.5)		21(4.9)	30(7.1)
Item8	142(33.4)	143(33.6)		10(2.4)	16(3.8)
Item9	184(43.3)	188(44.2)		14(3.3)	25(5.9)
Item10	106(24.9)	106(24.9)		21(4.9)	30(7.1)
Total score	1 (0.2)	2 (0.5)		1 (0.2)	1 (0.2)

## Discussion

According to the criterion of disease severity in psoriasis management guidelines [5], the BSA, PASI, DLQI and Index-16 scores in this study all showed that most patients with psoriasis suffer from moderate to severe effects of the disease. Further analysis revealed that the proportion of patients who chose moderate impact among the ten items of the DLQI ranged from 14.4 to 32.5%, indicating that disease had a moderate impact on all aspects of patients’ lives in a large proportion of patients. According to the criteria for the clinical classification of psoriasis severity, the DLQI-NS allowed 17 additional patients (4.0%) to achieve severe disease (PASI > 10 or BSA > 10% and DLQI > 10) [5]. The DLQI-NS may be useful when treatment plans are involved. In this study, both questionnaires showed overall good psychometric properties in psoriasis patients but the construct scores on DLQI-NS were superior to those on the DLQI. Additionally, no ceiling effects were found for any of the items of either the DLQI-NS or the DLQI scales.

In our study, the results of the patients’ QLQI scores indicated that disease had a moderate impact on all aspects of life in a large proportion of patients. This finding is similar to those of other studies [25, 26]. Belachew, E. A. reported that the QoL of approximately three-fourths of psoriasis patients was affected, and the severity of psoriasis ranged from very large to extremely large [26]. Psoriasis has a negative impact on the quality of life, especially in single and younger patients [25]. However, Ahmad Fuat MS reported that approximately 17% of patients experienced severe effects of psoriasis on their QoL [1]. Due to all participants were included in the inpatient department in our study, whereas other studies were conducted in outpatient settings or in the community. Generally, the severity of the disease is greater in inpatient departments than in outpatient departments. The total DLQI-NS score increased 17 patients (4.0%) to achieve a severe psoriasis rating (PASI > 10 or BSA > 10% and DLQI > 10) [5]. Treatment will not be delayed because the disease severity is insufficient. This effect is similar to that of the DLQI-R, but our scoring method is simpler [20]. The DLQI-NS score method used in this study is consistent with the original DLQI scale [5]. This may more accurately reflect the impact of the disease on a patient, especially in terms of moderate impact. Dermatologists may be more efficient if they use the DLQI-NS to assess patients’ quality of life and DLQI-NS may be useful when treatment plans are involved.

Both the DLQI-NS and DLQI showed overall good psychometric properties in psoriasis patients in this study. Both the DLQI-NS and DLQI have good reliability. The Cronbach’s alpha coefficient and test–retest reliability coefficient of both questionnaires were only slightly different. The Spearman-Brown coefficients and Guttman’s half coefficients of the DLQI-NS are better than those of the QLQI. The test–retest reliabilities and the split-half reliabilities of both questionnaires were excellent which were superior to those of Xiao Y’s study [27]. Xiao Y et al. [27] assessed the DLQI in a homogeneous population under lifetime arsenic exposure and found that the Cronbach’s alpha was 0.79 and that the split-half reliability was 0.77. This may be because the two scoring methods used in our study maintained a high degree of consistency.

The DLQI-NS scoring method increased only the moderate influence options in six dimensions of the original scale, namely, symptoms and feelings, daily activities, leisure, work and school, personal relationships, and treatment [26].

The DLQI-NS was superior to the DLQI in terms of construct validity according to the results of our study. Construct validity is a comprehensive judgment process that involves synthesizing various indicators, and its analysis has no fixed judgment criteria. Additionally, all items of the DLQI-NS and DLQI showed moderate to strong correlations with the total scores, which indicated that the structural validity of both questionnaires was good. The criterion validity of the DLQI-NS and DLQI were good. This was consistent with the results of the original scale [28]. However, few studies have shown the criterion validity of DLQI [14]. Both the DLQI-NS and DLQI were moderately to strongly correlated with the Skindex-16 in this study. This result is consistent with previous findings [16]. Interestingly, among the two DLQI correlation scales, the symptoms and feelings dimension had the lowest correlation coefficient with the emotions dimension of the Skindex-16, indicating a moderate correlation. Total scores had the strongest correlation with the functions dimension of the Skindex-16. It is possible that the DLQI, DLQI-NS and Skindex-16 are special scales for assessing skin diseases, so they are highly correlated and consistent in all aspects related to patients’ quality of life [12]. Regarding convergent validity, the total scores of the two scales were moderately correlated with the BSA and PASI in our study, which was consistent with previous findings [29]. Interestingly, neither scale correlated with the NRS score. These results contrast with those of a recent study with a homogeneous population [27]. Xiao’s study in a homogeneous population showed that the DLQI was positively correlated with the intensity of itching, as assessed by the NRS score [27]. This may be because the study populations were different. In our study, the itch score of the participants was mild and only some patients felt itching. However, the results were similar to those of Szabo et al., although the subjects were different [12].

No ceiling effects were found for any of the items of either the DLQI-NS or the DLQI scales. If more than 15% of respondents achieved the lowest or highest possible score, floor or ceiling effects, respectively, were considered to exist [30]. Eight items of both questionnaires exhibited high floor effects. Interestingly, floor effects were found for the same items on both questionnaires, which was different from the findings of recent studies [12]. The relatively high floor effect might lead to content validity issues, indicating that DLQI items might not effectively capture mild HRQoL problems. Further improvement studies of the DLQI may be needed to better capture problems related to quality of life in patients with skin diseases.

Our study has a few limitations. First, the DLQI-NS did not change the content of each item of the DLQI, so we did not validate the content validity. Because the DLQI items have been validated by dermatologists worldwide. Second, although the participants were located in western and southern China, but the study was conducted in only one center in China. Multicenter studies should be conducted to further demonstrate the value of the DLQI-NS for assessing HRQoL in psoriasis patients. Third, our study was a cross-sectional study without follow-up. A follow-up study is needed to validate our findings. In the next step, we will perform a multicenter study to validate the DLQI-NS.

## Conclusion

The DLQI-NS is an effective self-assessment tool for assessing skin disease quality of life. The validity and reliability of the DLQI-NS were superior to those of the DLQI. Moreover, these findings can more accurately reflect the extent of the disease’s impact on patients with psoriasis, especially in terms of moderate impact. It is more conducive to achieving the severe impact of disease severity classification stipulated in the guidelines and is conducive to the selection of treatment plans for patients. However, given the limited sample size of our study, further work is required to validate the effective application of DLQI-NS in a large cohort. There is currently little experience with using DLQI-NS, so, we encourage physicians to try out the DLQI-NS scoring chart and further discuss the clinical application of this method.

## Data Availability

No datasets were generated or analysed during the current study.

## Abbreviations

DLQI: Dermatology life quality index
DLQI-NS: DLQI new scoring method
PASI: Psoriasis area and severity index score
BSA: Body surface area score
NRS: Numerical rating scale

## References

[1] Ahmad Fuat MS, Mat Yudin Z, Muhammad J, Mohd Zin F. Quality of Life and Its Associated Factors among Patients with Psoriasis in a Semi-Urban Northeast Malaysia. International Journal of Environmental Research and Public Health.2022; 19(18). 10.3390/ijerph191811578 PMC9517003 36141851

[2] Nabieva K, Vender R (2023). Quality of life and body region affected by psoriasis: a systematic review. Actas Dermosifiliogr.

[3] Parisi R, Iskandar IYK, Kontopantelis E, Augustin M, Griffiths CEM (2020). National, regional, and worldwide epidemiology of psoriasis: systematic analysis and modelling study. BMJ.

[4] Thai S, Barlow S, Lucas J, Piercy J, Zhong Y (2023). Suboptimal clinical and quality of life outcomes in patients with psoriasis undertreated with oral therapies: International Physician and Patient Survey. Dermatol Ther (Heidelb).

[5] Nast A, Smith C, Spuls PI, Avila Valle G, Bata-Csorgo Z (2020). EuroGuiDerm Guideline on the systemic treatment of psoriasis vulgaris - part 1: treatment and monitoring recommendations. J Eur Acad Dermatol Venereol.

[6] Ali FM, Cueva AC, Vyas J, Atwan AA, Salek MS (2017). A systematic review of the use of quality-of-life instruments in randomized controlled trials for psoriasis. Br J Dermatol.

[7] van der Kraaij GE, Balak DMW, Busard CI, van Cranenburgh OD, Chung Y (2019). Highlights of the updated Dutch evidence- and consensus-based guideline on psoriasis 2017. Br J Dermatol.

[8] Gisondi PFM, Amerio P, Argenziano G, Bardazzi F, Bianchi L, Chiricozzi A, Conti A, Corazza M, Costanzo A, Dapavo P, DE Simone C, Fabbrocini G, Feliciani C, Foti C, Girolomoni G, Guarneri C, Marzano AV, Micali G, Offidani A, Parodi A, Pellacani G, Piaserico S, Prignano F, Romanelli M, Rongioletti F, Rubegni P, Stinco G, Stingeni L, Tomasini CF, Venturini M, Peris K, Calzavara-Pinton P (2022). Italian adaptation of EuroGuiDerm guideline on the systemic treatment of chronic plaque psoriasis. Ital J Dermatol Venerol.

[9] Kiltz U, Andreica I, Igelmann M, Kalthoff L, Krause D (2021). [Standardized documentation of health-related quality of life in patients with psoriatic arthritis: validation of the German version of the psoriatic arthritis quality of life (PsAQoL) questionnaire]. Z Rheumatol.

[10] Mease PJ, Deodhar AA, van der Heijde D, Behrens F, Kivitz AJ (2022). Efficacy and safety of selective TYK2 inhibitor, deucravacitinib, in a phase II trial in psoriatic arthritis. Ann Rheum Dis.

[11] Kumar A, Bhuyan D, Barua S, Dihingia S, Borgohain L. Psychiatric Morbidities and Their Impact on Quality of Life in Patients With Psoriasis. Cureus.2023. 10.7759/cureus.43394 PMC10495268 37706139

[12] Szabo A, Brodszky V, Rencz F (2022). A comparative study on the measurement properties of Dermatology Life Quality Index (DLQI), DLQI-Relevant and Skindex-16. Br J Dermatol.

[13] Al-Mazeedi K, El-Shazly M, Al-Ajmi HS (2006). Impact of psoriasis on quality of life in Kuwait. Int J Dermatol.

[14] Rencz F, Szabo A, Brodszky V (2021). Questionnaire modifications and alternative scoring methods of the Dermatology Life Quality Index: a systematic review. Value Health.

[15] Chernyshov PV (2019). The evolution of quality of Life Assessment and Use in Dermatology. Dermatology.

[16] Gergely LH, Gaspar K, Brodszky V, Kinyo A, Szegedi A (2020). Validity of EQ-5D-5L, Skindex-16, DLQI and DLQI-R in patients with hidradenitis suppurativa. J Eur Acad Dermatol Venereol.

[17] Menter A, Strober BE, Kaplan DH, Kivelevitch D, Prater EF (2019). Joint AAD-NPF guidelines of care for the management and treatment of psoriasis with biologics. J Am Acad Dermatol.

[18] Imafuku S, Zheng M, Tada Y, Zhang X, Theng C (2018). Asian consensus on assessment and management of mild to moderate plaque psoriasis with topical therapy. J Dermatol.

[19] Committee on Psoriasis CSoD (2023). Guideline for the diagnosis and treatment of psoriasis in China(2023 edition). Chin J Dermatol.

[20] Rencz F, Gulacsi L, Pentek M, Poor AK, Sardy M (2018). Proposal of a new scoring formula for the Dermatology Life Quality Index in psoriasis. Br J Dermatol.

[21] Rencz F, Gulacsi L, Pentek M, Szegedi A, Remenyik E (2020). DLQI-R scoring improves the discriminatory power of the Dermatology Life Quality Index in patients with psoriasis, pemphigus and morphea. Br J Dermatol.

[22] He Z, Lu C, Chren M-M, Zhang Z, Li Y et al. Development and psychometric validation of the Chinese version of Skindex-29 and Skindex-16. Health Qual Life Outcomes.2014; 12(1). 10.1186/s12955-014-0190-4 PMC4305222 25539748

[23] Henseler T, Schmitt-Rau K (2008). A comparison between BSA, PASI, PLASI and SAPASI as measures of disease severity and improvement by therapy in patients with psoriasis. Int J Dermatol.

[24] Phan N, Blome C, Fritz F, Gerss J, Reich A (2012). Assessment of Pruritus Intensity: prospective study on validity and reliability of the Visual Analogue Scale, Numerical Rating Scale and Verbal Rating Scale in 471 patients with chronic Pruritus. Acta Derm Venereol.

[25] Sendrasoa FA, Razanakoto NH, Ratovonjanahary V, Raharolahy O, Ranaivo IM et al. Quality of Life in Patients with Psoriasis Seen in the Department of Dermatology, Antananarivo, Madagascar. Biomed Res Int.2020; 2020, 9292163. 10.1155/2020/9292163 PMC7512037 33015185

[26] Belachew EA, Chanie GS, Gizachew E, Sendekie AK. Health-related quality of life and its determinants among patients with psoriasis at a referral hospital in Northwest Ethiopia. Front Med.2023; 10. 10.3389/fmed.2023.1183685 PMC10373881 37521356

[27] Xiao Y, Huang X, Jing D, Huang Y, Zhang X (2018). Assessment of the Dermatology Life Quality Index (DLQI) in a homogeneous population under lifetime arsenic exposure. Qual Life Res.

[28] Takahashi N, Suzukamo Y, Nakamura M, Miyachi Y, Green J et al. Japanese version of the Dermatology Life Quality Index: validity and reliability in patients with acne. Health Qual Life Outcomes.2006; 4(1). 10.1186/1477-7525-4-46 PMC1557837 16884543

[29] Barbieri JS, Gelfand JM (2019). Evaluation of the Dermatology Life Quality Index scoring modification, the DLQI-R score, in two independent populations. Br J Dermatol.

[30] Terwee CB, Bot SDM, de Boer MR, van der Windt DAWM, Knol DL (2007). Quality criteria were proposed for measurement properties of health status questionnaires. J Clin Epidemiol.

